# Interdisciplinary care clinics in chronic kidney disease

**DOI:** 10.1186/s12882-015-0158-6

**Published:** 2015-10-12

**Authors:** Tanya S. Johns, Jerry Yee, Terrian Smith-Jules, Ruth C. Campbell, Carolyn Bauer

**Affiliations:** 1 Department of Medicine, Division of Nephrology, Albert Einstein School of Medicine, Montefiore Medical Center, 111 East 210th Street, Bronx, NY 10467 USA; 2 Department of Internal Medicine, Division of Nephrology and Hypertension, Henry Ford Hospital, Detroit, MI USA; 3 Care Management Organization, Montefiore Medical Center, Yonkers, NY USA; 4 Department of Medicine, Division of Nephrology, Medical University of South Carolina, Charleston, SC USA

**Keywords:** Interdisciplinary care, Patient-centered, Quality improvement, Clinical outcomes

## Abstract

The burden of chronic kidney disease (CKD) is substantial, and is associated with high hospitalization rates, premature deaths, and considerable health care costs. These factors provide strong rationale for quality improvement initiatives in CKD care. The interdisciplinary care clinic (IDC) has emerged as one solution to improving CKD care. The IDC team may include other physicians, advanced practice providers, nurses, dietitians, pharmacists, and social workers—all working together to provide effective care to patients with chronic kidney disease. Studies suggest that IDCs may improve patient education and preparedness prior to kidney failure, both of which have been associated with improved health outcomes. Interdisciplinary care may also delay the progression to end-stage renal disease and reduce mortality. While most studies suggest that IDC services are likely cost-effective, financing IDCs is challenging and many insurance providers do not pay for all of the services. There are also no robust long-term studies demonstrating the cost-effectiveness of IDCs. This review discusses IDC models and its potential impact on CKD care as well as some of the challenges that may be associated with implementing these clinics.

## Background

### Defining the need for interventions in the chronic kidney disease population

Chronic kidney disease, defined as persistent albuminuria or glomerular filtration rate (GFR) <60 ml/min/1.73 m^2^, affects ~12 % of the US population [1]. Individuals with CKD are at increased risk for hospitalizations, cardiovascular events, and mortality [2, 3]. The CKD population is burdened with socioeconomic challenges including high poverty rates [4] and low health literacy [5], which contribute to poor outcomes [6, 7].

Areas for improvement in CKD care include patient education, management of CKD risk factors, and complications, and timely patient preparation for ESRD. Nephrologists must also contend with a complex patient population, increasing workload, and the pressure for improved outcomes with a shrinking workforce that is associated with fewer fellowship applicants [8]. These issues have led to a critical appraisal of how CKD care is being delivered and what outcomes should be followed in health care systems. Interdisciplinary care clinic have emerged as an alternative to traditional nephrology care in response to many of these issues. Such clinics address quality improvement, CKD management, and patient education. Our intent is to discuss the potential role of IDCs on improving CKD health outcomes in the United States. We reviewed existing literature on CKD care in PubMed using key words “interdisciplinary”, “multidisciplinary”, or “coordinated” to find articles relevant to the topic.

## Review

### Definition and domains of interdisciplinary care clinics

**Fig. 1:**
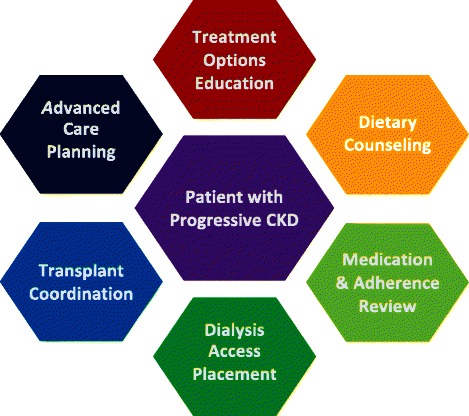
Domains of interdisciplinary chronic kidney disease care

Interdisciplinary care is a coordinated, patient-centered approach that integrates separate disciplines to achieve common management goals [9]. Patients are empowered to be part of the decision-making process, including the setting of short- and long-term goals. There is no single description of what constitutes an IDC in CKD, which at a minimum should provide coordinated patient-centered care that addresses meaningful CKD education and effectively prepares patients for end-stage renal disease (ESRD). The KDIGO 2012 guidelines specify that interdisciplinary nephrology care should encompass patient education regarding different renal replacement therapy (RRT) modalities and transplantation, dietary counseling, early vascular access placement, and ethical, psychological and social care [10] (Fig. 1). This approach to CKD care often entails nephrologists and other health care providers from different disciplines (e.g., other physicians, advanced practice professionals (APP) formerly known as physician extenders, pharmacists, social workers, and dietitians) collaboratively implementing evidence-based guideline-driven protocols in CKD care within the confines of the patient’s (or proxy’s) expressed wishes (Table 1).

**Table 1:** Potential roles for an interdisciplinary care clinic in CKD (modeled on montefiore medical center kidney care program)

Nephrologist	Evaluates etiology of CKD and determines the care plan
Advanced practitioner	Educates about CKD and kidney failure treatment options
	Coordinates care with family and members of the IDC team
Dietitian	Dietary counseling and fluid management
Pharmacist	Reviews medications, dosing, and adherence
	Educates patients about the use of over the counter medications and herbal preparations
Geriatrician/palliative care	Addresses geriatric and palliative care needs
	Discusses prognosis and ensures treatment plans align with goal of care
Case management/social work	Assists patients to obtain needed resources (e.g., transportation and issues with housing)
Transplant team	Educates patients about transplant options
	Evaluates potential transplant candidates with progressive CKD
Vascular surgery/general surgery	Places and monitors access for dialysis (hemodialysis and peritoneal dialysis)
Interventional radiology	Intervenes on immature or nonfunctioning AVG/AVF to improve access flow in order to initiate dialysis

*IDC* interdisciplinary care clinic, *AVG* arteriovenous graft, *AVF* arteriovenous fistula

One of the most important domains of IDCs in CKD care is to provide patient education. Despite decades of awareness regarding the importance of patient education and preparedness in improving health outcomes in CKD [11, 12], national indicators demonstrate that predialysis education is unacceptably low in the CKD population and may not be optimal for maximizing dialysis preparedness. For those patients that choose hemodialysis, one surrogate of education and preparedness is the timely placement of permanent dialysis vascular access. Almost 80 % of newly initiated hemodialysis (HD) patients begin dialysis with a catheter [13]. Early referral to a nephrologist is necessary [14] but not sufficient for improving this outcome parameter. A significant number of patients who are followed by a nephrologist before initiating RRT will still start with temporary vascular access; a lack of education is likely a part of this phenomenon [15, 16]. Low health literacy has been shown to affect permanent vascular access placement in ESRD patients [17].

Predialysis education is the most studied aspect of IDC in nephrology. It is associated with increased selection of home HD and peritoneal dialysis (PD) modalities, improved permanent access placement and reduced mortality [17, 18]. Educational interventions offered for only one day have been shown to have significant benefits [11, 19]. In 2010, under the Medicare Improvement of Patients and Providers Act (MIPPA), Medicare started to reimburse for CKD education provided by a physician, APP, or clinical nurse specialist for Medicare recipients with CKD stage 4 or 5. Up to six sessions of education are reimbursed, and may be delivered either as a class or on an individual basis in the outpatient setting [20]. The classes may cover management of comorbidities, prevention of uremic complications, and options for RRT (in-center HD, home therapies including PD, access options and transplantation). A recent survey of US nephrology practices found that only about 60 % offered a CKD education class and that an advanced practitioner delivered 87 % of the classes [21]. While CKD education is most commonly conducted in the outpatient setting, education may also be delivered effectively in the hospital. Rioux et al. described a program of CKD education for hospitalized patients who needed to start dialysis acutely. Their intervention, an APP providing 3–5 inpatient sessions and a multimedia presentation, including handouts and DVDs, resulted in 31 % of patients choosing a home dialysis modality prior to hospital discharge [22].

An important consideration in CKD education programs is the impact of health literacy. Low health literacy, or how well a patient understands and assimilates information to make decisions regarding his or her health, is common in the US and among CKD patients [23]. It is associated with increased mortality on dialysis [7] and with lower referral rates for transplantation [24]. These data suggest that both education and assessment of patients’ understanding of their disease and potential treatments are essential to provide the most effective CKD care.

Another important domain of IDCs is establishment of patient-centered goals of care. This is particularly important among high-risk groups. Among the elderly, for example, the high risk of mortality and decreased functional status associated with ESRD [25, 26] warrants advanced care planning as part of the services provided by IDCs. In the US, 20 % of patients who died in the initial 120 days after starting dialysis stopped treatment [27]. Planning ahead and clarifying patients’ wishes prior to ESRD may decrease aggressive and costly measures. An elderly patient may choose a time-trial of dialysis with specific withdrawal parameters. Geriatric-palliative care physicians, as part of the interdisciplinary care team, may assess patients’ functional and decision-making capacities and help determine and communicate overall prognosis for the elderly. The social workers in the team could assist with supplying resources, the completion of health care proxy and advanced directives (Table 1). For patients who decide on less aggressive medical management of their stage 5 CKD—the “no dialysis” option—IDC programs should continue to provide services that facilitate patient overall wellness and comfort.

### Goals of interdisciplinary care clinics

The ultimate goals of IDCs are to improve morbidity and mortality for patients with CKD. To achieve these goals, IDCs will need to focus on managing cardiovascular risks, implement practices to retard the progression of CKD, and transition patients safely from CKD to ESRD. Other common goals of IDCs are to identify and manage the complications of CKD such as hypertension, anemia, mineral and bone disorders, electrolyte disturbances and fluid imbalances according to guidelines established by Kidney Disease Quality Initiative Outcomes (KDIGO) [10, 28]. IDCs may also support vaccination against influenza, *Streptococcus pneumoniae*, and hepatitis B given the immunosuppressive nature of CKD [29, 30].

#### IDC in improving morbidity and mortality

Traditional risk factors for CVD such as increasing age, hypertension, diabetes, and hyperlipidemia are highly prevalent in the CKD population [31]. Lipid-lowering therapies in CKD patients have been shown to improve cardiovascular outcomes [32, 33]. Other nontraditional risk factors for CVD have been identified in patients with CKD, including albuminuria, anemia, fluid overload, vascular calcification, inflammation, malnutrition, and increased oxidative stress [34–37]. Dietitians, with renal-specific training, in an IDC model should work with patients to prevent, diagnose, and address malnutrition. Smoking is associated with CVD and observational data suggest that smoking may influence the progression of renal disease, and therefore, smoking cessation should to be addressed in CKD patient [38, 39].

#### IDC in slowing CKD progression

CKD clinics should strongly advocate for the use interventions that slow the decline of renal function as feasible. Unfortunately, very few interventions have been shown to slow progression of CKD. Control of hypertension, especially with angiotensin converting enzyme inhibitors or angiotensin receptor blockers in diabetics and non-diabetics with proteinuria could delay the progression of CKD [40–44] and reduce cardiovascular events [45]. Other interventions that may slow progression of kidney disease include the treatment of metabolic acidosis, avoiding nephrotoxins (e.g., nonsteroidal anti-inflammatory drugs) and effective patient education [46, 47]. Pharmacists in an IDC may work in concert with nephrologists to ensure patient medication adherence, and to determine inappropriate medications that should be discontinued or medications that need to be re-dosed to patients’ GFRs. Education also plays a role in delaying ESRD. One randomized study of advanced CKD patients revealed that a single education session with nurse follow-up was associated with delay in the initiation of RRT by approximately 3 months [48].

#### IDC in transitioning patients from CKD to ESRD

Another important goal of IDCs is to safely and effectively transition patients with advancing CKD to ESRD care. In particular, the IDC should aim to lower the high morbidity and mortality associated with the commencement of dialysis [13, 49]. A planned transition to dialysis should result in improved health and reduced costs for patients. The IDC team should help to create patient-centered action plans for initiating dialysis, coordinate listing for kidney transplantation, and define goals of care for patients who may not desire or benefit from these modalities. Education, in individual or group sessions, regarding RRT is paramount to this process. Patients (and their families) must understand the different options RRT (PD, in-center and home HD, and transplantation) to facilitate informed decision-making that best accommodates patients’ desires and circumstances. A discussion of an individual’s prognosis and the risks and benefits of dialysis and transplantation should be provided to each patient. Preparation for dialysis should occur simultaneously with evaluation for transplantation. Ideally, the modality for RRT should be established at least 6 months to 1 year prior to the development of ESRD to allow for timely access placement and the work-up required prior to transplantation. Patients beginning dialysis via a HD catheter have a significantly increased risk of death compared to patients who began dialysis with an arteriovenous fistula (AVF) [50, 51]. Partnership with a vascular surgeon with high-level expertise in AVFs and arteriovenous grafts (AVGs) construction would ensure consistent establishment of functioning AVFs in suitable individuals. As starting HD in the hospital is costly, interdisciplinary CKD programs should aim to facilitate outpatient dialysis starts in appropriate patients. Patients should be given options to choose home modalities (such as PD and home HD) because these modalities preserve independence and quality of life better than in-center hemodialysis and are less costly. Transplantation prior to starting RRT is associated with improved patient and kidney allograft survival [52–54]. The IDC may coordinate with a transplant center to ensure timely referrals to transplantation, transplantation education, and discussions between patients and their families regarding live kidney donation.

### Models of IDCs and barriers to interdisciplinary care

The most common IDC structure is to have interdisciplinary services provided by the nephrology practice. Instead of being embedded in the practice, some IDCs may run in parallel to a nephrology practice, with patients alternating between IDC visits and general nephrology visits. In this model, it is imperative that the IDC and nephrologist work in unison as a team and not as competing providers. Another structural component to consider is who is referring the patients to the IDC. Some IDCs may incorporate a nephrology evaluation as part of their services and take referrals directly from primary care physicians (PCPs) [55], while other clinics may offer only education or protocol driven management of CKD and not accept a referral from a PCP. This illustrates the importance of clearly defining the role of the IDC in relation to both nephrology and primary care.

Financial constraints of the providers and patients may limit the ability to offer all services to patients. As such, nephrology practices may seek to identify and prioritize the services that are needed the most by their constituent populations to improve health outcomes. In order to achieve this, the CKD clinic team may implement quality improvement processes to monitor outcomes and maximize patient benefits. Patient-level barriers such as education attained, health literacy, family support, and psychosocial and cultural beliefs may also affect the implementation and effectiveness of IDCs. The additional time spent in IDCs and potential additional cost incurred by patients in travel may overwhelm some and could adversely impact patient engagement. Overcoming some of these barriers may necessitate intensive case management and are potentially costly.

### Economics of interdisciplinary care clinics

Each IDC is setup differently and influenced by variables such as budgeting and office space. Funding for IDCs may vary greatly in different countries depending on the resources of the national health care system and the extent of health care privatization. In the United States, APPs can bill most insurance companies directly, but the standard fee-for-service model may not reimburse some of the other team members. Renal dietitians are reimbursed by Medicare for stage 4 CKD patients, but other insurance companies may not cover their services. A social worker is reimbursed for services only when providing counseling for a Diagnostic and Statistical Manual of Mental Disorders (DSM)-5 diagnosis. Pharmacist services are not reimbursable. These financial constraints may make it difficult for nephrologists to provide an IDC model of CKD to their patients. Grants, awards, donations, or alternative sources of funding may be required, which could limit IDCs in the US to large academic institutions. The creation of accountable care organizations (ACOs), which are groups of health care providers that assume responsibility for the quality, cost-effectiveness, and general health care of specified US Medicare beneficiaries, may represent an important source of funding for these IDC initiatives in the future.

Increased outpatient dialysis starts and greater AVF and PD selection rates may help persuade funding sources that CKD clinics are cost-effective and sustainable. In 2007, the average inpatient cost for the first month of dialysis in the U.S. was $9,846 per Medicare member and $22,841 per employer group health plan member [56]. These costs could be greatly reduced by increasing outpatient dialysis starts. Patients with AVFs compared to those with HD catheters had a lower total per member per year cost at $64,701 and $90,110, respectively [56]. Increasing rates of PD as the initial modality for ESRD is also cost-effective. According to the United States Renal Data System database, the total yearly expenditures per patient in 2010 for HD was $87,561 compared to $66,751 for PD, a savings of over $20,000 per patient per year [13]. Overall, the cost savings to be gained through an improved transition to ESRD likely outweigh the greater costs of an IDC team.

### Target population for interdisciplinary care clinics

Patients at the highest risk for progression to ESRD or patients who have complications of CKD that need specialized management (e.g., anemia and mineral bone disorders) are logical populations to target. The 2012 KDIGO CKD guidelines suggest that patients with progressive CKD be treated in an IDC setting, but the guidelines do not specify a GFR cutoff [28]. Therefore, identification of patients at greatest risk is necessary. A number of validated prediction models have been developed to help estimate the risk of progression to ESRD [57]. Tangri et al. developed a prediction model that uses age, sex, estimated glomerular filtration rate (eGFR), urine albumin-to-creatinine ratio, calcium, phosphorus, albumin and bicarbonate to predict risk of progression [58], which is also available as a software application for smart devices (http://www.qxmd.com/specialty/medicine/nephrology-medical-apps-iphone-blackberry-android). More recently, Maziarz et al. showed that among the urban poor, a prediction model using five common variables (age, sex, race, eGFR, and dipstick proteinuria) performed similarly to more complex models that incorporated extensive sociodemographic and clinical data [59].

### Clinical outcomes of interdisclipinary clinics in chronic kidney disease care

While the variety of structures makes it difficult to study outcomes and efficacy of IDCs in CKD care, the literature does suggest that these programs improve AV access placement before initiation of RRT, reduce mortality rates during the transition from CKD to ESRD, slow progression of CKD, and are likely cost-effective.

One study by Snyder and Collins found that a higher number of preventive measures including monitoring lipids, glucose control, and mineral and bone parameters as well as influenza vaccination were associated with low rates of atherosclerotic heart disease in a Medicare CKD cohort [60]. In a Canadian province, Hemmelgarn et al. demonstrated a 50 % reduction in risk of death in a predialysis cohort who received interdisciplinary care compared with a propensity-matched control group who had usual nephrology care (Table 1) [61]. A prospective cohort study in Taiwan also demonstrated decreased mortality for those patients who underwent IDC care [62]. Two other cohort studies supported that exposure to IDC care decreased mortality rates after dialysis initiation [12, 63]. Fresenius Medical Care North America, a large dialysis organization, established that patients who underwent a predialysis educational program were significantly more likely to choose PD as their RRT modality, begin HD with an AVF or AVG, and less likely to die within the first 90 days following onset of dialysis [64]. The Multifactorial Approach and Superior Treatment Efficacy in Renal Patients with the Aid of Nurse Practitioners (MASTERPLAN), a randomized trial of 788 Dutch patients, showed that the additional implementation of current CKD guidelines by APPs in CKD stage 3 and 4 patients compared to usual nephrology care slowed their rates of decline of eGFR. Also, the risk of developing the composite end-point of death, ESRD and a 50 % increase in serum creatinine over a median follow-up of six years was reduced [65]. The previously published MASTERPLAN trial at a median follow-up of 5 years did not reveal a significant difference in CVD outcomes but showed that APP supported care decreased CVD risk factors (high blood pressure, LDL cholesterol, anemia, and proteinuria) [66] (Table 2). The generalizability of this study may be limited by the inclusion of a relatively young and “healthy” CKD patient population. Although the study was not designed to evaluate the cost-effectiveness of the intervention, the authors reported a crude estimate of savings and costs, implying cost benefits of the MASTERPLAN study [65].

**Table 2:** Studies Comparing Interdisciplinary Care Models to Standard Nephrology Care for Mortality, Hospitalizations and Renal Outcomes

Study, year	Study population and design	Exposure or intervention	Outcomes	Major findings	Cost-benefit
Curtis et al., 2003 [63]	Retrospective cohort study of 288 incident dialysis patients (mean age 62 years) in Canada and Italy	Formalized multidisciplinary clinic programs consisting of a nurse educator, physician, social worker, nutritionist, and pharmacist	Mortality up to 2.5 years after dialysis initiation	HR 0.46 (95 % CI 0.23–0.90) for IDC group after adjustments for age, sex, calculated GFR at dialysis start, race, diabetes, etiology of kidney failure, and country of treatment	Not assessed
Goldstein et al., 2004 [12]	Retrospective cohort study of 184 Canadian incident dialysis patients (mean age 60 years)	Progressive multidisciplinary renal disease clinic that included a dietitian, nurse educator, pharmacist, social worker and volunteer peer supporters	Mortality and hospitalizations at 1 year after starting dialysis	Fewer deaths in the IDC group (2 % versus 23 %; *P <* 0.01) and fewer hospitalizations (7 versus 69.7 days/patient/year (*P* < 0.01)	Not assessed
				Independent predictors of death were older age, history of cardiovascular disease and non-IDC.	
Hemmelgarn et al., 2009 [61]	Propensity score matched cohort study of 6978 elderly Canadian patients (mean age 76 years) with CKD stage 4 and 5	Multidisciplinary care clinic utilizing nurses, dietitians and social workers	1. Mortality 2. All-cause and cardiovascular-specific hospitalizations	HR 0.50 (95 % CI 0.35–0.71) for the IDC group after adjustments for age, gender, baseline GFR, diabetes, and comorbidity score in the MDC group compared to standard group No difference in all-cause (HR 0.83; 95 % CI 0.64– 1.06) or cardiovascular-specific hospitalization (HR 0.76; 95 % CI 0.54 to 1.06) adjusted for age, gender, baseline GFR, diabetes, and comorbidity score	Not assessed
Wu et al., 2009 [62]	Prospective cohort study of 573 Taiwanese patients (mean age 63 years) with GFR <60 ml/min/1.73 m^2^	Multidisciplinary care with nurses for case management, dietitians, volunteer peer supporters	1. Progression to ESRD	HR 0.117 (95 % CI 0.075–0.183) for the IDC group after adjustments for age, gender, DM and HTN status, baseline eGFR, hemoglobin and albumin	Not assessed
			2. All-cause mortality	HR 0.10 (95 % CI 0.04–0.265) for the IDC group after adjustments for gender, DM and HTN status, baseline eGFR, hemoglobin and albumin	
Wei et al., 2010 [71]	Cohort study of 137 Taiwanese patients (mean age 57 control group and 63 exposed group) with CKD stage 3–5	Multidisciplinary team including renal nurses and dieticians	Hospitalization for hemodialysis initiation	40.8 % in the intervention group were not hospitalized compared to 18.8 % in the usual care group (*P* < 0.005)	Favored intervention
Lacson et al., 2010 [64]	Matched (1:1) study of 2,800 incident dialysis (mean age 63 years) in the United States	Educational program on treatment options for dialysis	Mortality within the first 90 days of starting dialysis	HR 0.61 (95 % CI 0.50–0.74) for treatment options attendees compared to usual care after adjustments for case-mix and laboratory data	Not assessed
Barrett et al., 2011 [69] CanPREVENT	Randomized control trial of 474 patients (mean age 67 years) with CKD stage 3 and 4 in Canada	Nurse-coordinated care focused on risk factor modification	Rate of decline in GFR	Nurse-coordinated team did not alter rate of GFR decline	Not assessed
Baylis et al., 2011 [68]	Cohort study of 2002 patients (mean age 68 years) with CKD stage 3 in the United States	Multidisciplinary team consisting of nephrologist, renal clinical pharmacy specialist, diabetes nurse educator, renal dietitian, social worker, and nephrology nurse	Rate of decline in GFR	Mean annual decline in GFR 1.73 ml/min/1.73 m^2^ in the intervention group compared to 2.1 ml/min/1.73 m^2^ in the usual care group after adjustments for nephrology site, follow-up time, race, age, baseline GFR, gender, number of chronic conditions, body mass index, number of GFR measurements, and number of primary care visit *(P* < 0.0001*)*	Not assessed
Devins et al., 2011 [48]	Multi-center randomized control trial of 323 Canadian patients (mean age 54 years) with progressive CKD (deemed likely start dialysis in next 6 to 12 months)	Predialysis psychoeducation	Time to dialysis initiation	Median time to dialysis was 17.0 months in the intervention group compared to 14. 2 months in usual-care control group (*P* < 0.001)	Not assessed
Van Zullen et al., 2012 [66] MASTERPLAN	Randomized control trial of 788 patients (mean age 59 years) from the Netherlands with CKD stage 3 and 4	Addition of nurse practitioner coordinated care	1. Composite of myocardial infarction, stroke, or cardiovascular death.	No difference (HR 0.90; 95 % CI 0.58–1.39)	
			2. Composite vascular interventions, all-cause mortality or end-stage renal disease	No difference (HR 0.83; 95 % CI 0.57–1.20)	
Peeters et al., 2014 [65] MASTERPLAN			1. Composite of incident ESRD, death, or 50 % increase in creatinine	HR 0.80 (95 % CI 0.66–0.98) in the intervention group vs. control	Crude estimate of savings and costs favored intervention
			2. Difference in slope of GFR	Decrease in estimated GFR was 0.45 ml/min per 1.73 m^2^ per year less in intervention group vs. control (*P* = 0.01)	

*HR* hazard ratio, *CI* confidence interval, *IDC* interdisciplinary care clinic

Similar to MASTERPLAN [65], other studies have also shown that IDC services may slow progression of CKD. A randomized trial demonstrated that a single 90-min education session along with follow-up phone calls significantly delayed dialysis initiation by approximately 3 months in patients expected to start dialysis within 6–18 months [48]. A program in England determined that patients with eGFRs less than 30 ml/min/1.73 m^2^who had access to a nurse, patient education, medication management, and nutrition counseling had a decreased rate of eGFR decline, with the greatest benefit to those patients with rapidly progressive CKD [67].

In early CKD and non-progressive CKD, there is conflicting data on the efficacy of IDC care. In a large health maintenance organization population, a study found a slower eGFR decline in those patients who were enrolled in IDCs compared with historical controls [68]. The Canadian Prevention of Renal and Cardiovascular Endpoints Trial (CanPREVENT) did not show that nurse-coordinated care improved the rate of GFR decline or control of most risk factors compared with usual care in patients with largely non-progressive kidney disease [69]. However, nurse-coordinated care entailed several benefits including fewer visits to specialists such as cardiologists and fewer days in hospital [69]. Therefore, nurse-coordinated care following stratification of CKD by stage as well as type may offer a cost-effective solution to the overall cost of health care.

The literature on interdisclipinary care services has more consistently demonstrated the increased use of arteriovenous accesses at HD initiation and decreased hospitalization rates. Cohort studies performed in California, Taiwan, and Canada demonstrated that patients exposed to IDC care had significantly decreased hospitalizations and more AVFs [19, 70, 71]. One single-center study revealed that guideline-driven care by APPs was associated with improved functioning, permanent vascular accesses and decreased hospitalizations 12 months after dialysis initiation [72].

## Conclusion

Interdisclipinary care clinics in CKD care are associated with greater patient preparedness and improved health outcomes during the transition from CKD to ESRD, especially among patients at increased risk for CKD progression based on risk of ESRD prediction, sociodemographic factors, eGFR level, and rate of decline of eGFR. While different models for IDC in CKD care exist, the goal should be to include those interventions with demonstrated success within the limitations of available resources. Although IDCs appear promising in CKD care, studies with longer follow-up and higher risk patients are required to better understand the quality and utility of IDCs. Funding for IDC services may be challenging despite the potential cost savings of such clinics. Therefore, robust studies regarding the cost-effectiveness of IDCs should be pursued, planned and performed.

## References

[1] Levey AS, Stevens LA, Schmid CH, Zhang Y, Castro AF, Feldman HI, Kusek JW, Eggers P, Van Lente F, Greene T (2009). A New Equation to Estimate Glomerular Filtration Rate. Ann Intern Med.

[2] U.S. Renal Data System, USRDS 2013 Annual Data Report: Atlas of Chronic Kidney Disease and End-Stage Renal Disease in the United States. National Institutes of Health, National Institute of Diabetes and Digestive and Kidney Diseases. Bethesa, MD; 2013. [[online] http://www.usrds.org/atlas.aspx]

[3] Go AS, Chertow GM, Fan D, McCulloch CE, Hsu CY (2004). Chronic kidney disease and the risks of death, cardiovascular events, and hospitalization. N Engl J Med.

[4] Crews DC, Charles RF, Evans MK, Zonderman AB, Powe NR (2010). Poverty, race, and CKD in a racially and socioeconomically diverse urban population. Am J Kidney Dis.

[5] Devraj R, Gordon EJ (2009). Health literacy and kidney disease: toward a new line of research. Am J Kidney Dis.

[6] Crews DC, McClellan WM, Shoham DA, Gao L, Warnock DG, Judd S, Muntner P, Miller ER, Powe NR (2012). Low Income and Albuminuria Among REGARDS (Reasons for Geographic and Racial Differences in Stroke) Study Participants. Am J Kidney Dis.

[7] Cavanaugh KL, Wingard RL, Hakim RM, Eden S, Shintani A, Wallston KA, Huizinga MM, Elasy TA, Rothman RL, Ikizler TA (2010). Low health literacy associates with increased mortality in ESRD. J Am Soc Nephrol.

[8] Parker MG, Ibrahim T, Shaffer R, Rosner MH, Molitoris BA (2011). The future nephrology workforce: will there be one?. Clin J Am Soc Nephrol..

[9] Jessup RL (2007). Interdisciplinary versus multidisciplinary care teams: do we understand the difference?. Aust Health Rev.

[10] Kidney Disease: Improving Global Outcomes (KDIGO) CKD Work Group (2013). KDIGO 2012 clinical practice guideline for the evaluation and management of chronic kidney disease. Kidney Int Suppl.

[11] Devins GM, Mendelssohn DC, Barre PE, Taub K, Binik YM (2005). Predialysis psychoeducational intervention extends survival in CKD: a 20-year follow-up. Am J Kidney Dis.

[12] Goldstein M, Yassa T, Dacouris N, McFarlane P (2004). Multidisciplinary predialysis care and morbidity and mortality of patients on dialysis. Am J Kidney Dis.

[13] U.S. Renal Data System (2012). USRDS 2012 annual data report: atlas of chronic kidney disease and end-stage renal disease in the United States.

[14] Smart NA, Dieberg G, Ladhani M, Titus T (2014). Early referral to specialist nephrology services for preventing the progression to end-stage kidney disease. Cochrane Database Syst Rev.

[15] Stehman-Breen CO, Sherrard DJ, Gillen D, Caps M (2000). Determinants of type and timing of initial permanent hemodialysis vascular access. Kidney Int.

[16] Buck J, Baker R, Cannaby AM, Nicholson S, Peters J, Warwick G (2007). Why do patients known to renal services still undergo urgent dialysis initiation? A cross-sectional survey. Nephrol Dial Transplant.

[17] Cavanaugh KL, Wingard RL, Hakim RM, Elasy TA, Ikizler TA (2009). Patient dialysis knowledge is associated with permanent arteriovenous access use in chronic hemodialysis. Clin J Am Soc Nephrol.

[18] Goovaerts T, Jadoul M, Goffin E (2005). Influence of a pre-dialysis education programme (PDEP) on the mode of renal replacement therapy. Nephrol Dial Transplant.

[19] Levin A, Lewis M, Mortiboy P, Faber S, Hare I, Porter EC, Mendelssohn DC (1997). Multidisciplinary predialysis programs: quantification and limitations of their impact on patient outcomes in two Canadian settings. Am J Kidney Dis.

[20] Young HN, Chan MR, Yevzlin AS, Becker BN (2011). The rationale, implementation, and effect of the Medicare CKD education benefit. Am J Kidney Dis.

[21] Davis JS, Zuber K (2013). Implementing patient education in the CKD clinic. Adv Chronic Kidney Dis.

[22] Rioux JP, Cheema H, Bargman JM, Watson D, Chan CT (2011). Effect of an in-hospital chronic kidney disease education program among patients with unplanned urgent-start dialysis. Clin J Am Soc Nephrol.

[23] Fraser SD, Roderick PJ, Casey M, Taal MW, Yuen HM, Nutbeam D (2013). Prevalence and associations of limited health literacy in chronic kidney disease: a systematic review. Nephrol Dial Transplant.

[24] Grubbs V, Gregorich SE, Perez-Stable EJ, Hsu CY (2009). Health literacy and access to kidney transplantation. Clin J Am Soc Nephrol.

[25] Kurella Tamura M, Covinsky KE, Chertow GM, Yaffe K, Landefeld CS, McCulloch CE (2009). Functional status of elderly adults before and after initiation of dialysis. N Engl J Med.

[26] Kurella M, Covinsky KE, Collins AJ, Chertow GM (2007). Octogenarians and nonagenarians starting dialysis in the United States. Ann Intern Med.

[27] Robinson BM, Zhang J, Morgenstern H, Bradbury BD, Ng LJ, McCullough KP, et al. Worldwide, mortality risk is high soon after initiation of hemodialysis. Kidney international Epub. 2013. 10.1038/ki.2013.252 PMC3877739 23802192

[28] National Kidney Foundation. KDOQI clinical practice guidelines for chronic kidney disease: evaluation,classification, and stratification. Am J Kidney Dis. 2002;39(2 Suppl 1):S1-266. 11904577

[29] Dalrymple LS, Katz R, Kestenbaum B, de Boer IH, Fried L, Sarnak MJ, Shlipak MG (2012). The risk of infection-related hospitalization with decreased kidney function. Am J Kidney Dis.

[30] Dinits-Pensy M, Forrest GN, Cross AS, Hise MK (2005). The use of vaccines in adult patients with renal disease. Am J Kidney Dis.

[31] Muntner P, He J, Astor BC, Folsom AR, Coresh J (2005). Traditional and nontraditional risk factors predict coronary heart disease in chronic kidney disease: results from the atherosclerosis risk in communities study. J Am Soc Nephrol.

[32] Tonelli M, Isles C, Curhan GC, Tonkin A, Pfeffer MA, Shepherd J, Sacks FM, Furberg C, Cobbe SM, Simes J (2004). Effect of pravastatin on cardiovascular events in people with chronic kidney disease. Circulation.

[33] Baigent C, Landray MJ, Reith C, Emberson J, Wheeler DC, Tomson C, Wanner C, Krane V, Cass A, Craig J (2011). The effects of lowering LDL cholesterol with simvastatin plus ezetimibe in patients with chronic kidney disease (Study of Heart and Renal Protection): a randomised placebo-controlled trial. Lancet.

[34] Matsushita K, van der Velde M, Astor BC, Woodward M, Levey AS, de Jong PE, Coresh J, Gansevoort RT (2010). Association of estimated glomerular filtration rate and albuminuria with all-cause and cardiovascular mortality in general population cohorts: a collaborative meta-analysis. Lancet.

[35] Jurkovitz CT, Abramson JL, Vaccarino LV, Weintraub WS, McClellan WM (2003). Association of high serum creatinine and anemia increases the risk of coronary events: results from the prospective community-based atherosclerosis risk in communities (ARIC) study. J Am Soc Nephrol..

[36] Muntner P, Hamm LL, Kusek JW, Chen J, Whelton PK, He J (2004). The prevalence of nontraditional risk factors for coronary heart disease in patients with chronic kidney disease. Ann Intern Med.

[37] Gosmanova EO, Le NA (2011). Cardiovascular Complications in CKD Patients: Role of Oxidative Stress. Cardiol Res Pract.

[38] Hallan SI, Orth SR (2011). Smoking is a risk factor in the progression to kidney failure. Kidney Int.

[39] Orth SR, Hallan SI (2008). Smoking: a risk factor for progression of chronic kidney disease and for cardiovascular morbidity and mortality in renal patients--absence of evidence or evidence of absence?. Clin J Am Soc Nephrol.

[40] Wright JT, Bakris G, Greene T, Agodoa LY, Appel LJ, Charleston J, Cheek D, Douglas-Baltimore JG, Gassman J, Glassock R (2002). Effect of blood pressure lowering and antihypertensive drug class on progression of hypertensive kidney disease: results from the AASK trial. JAMA.

[41] Agodoa LY, Appel L, Bakris GL, Beck G, Bourgoignie J, Briggs JP, Charleston J, Cheek D, Cleveland W, Douglas JG (2001). Effect of ramipril vs amlodipine on renal outcomes in hypertensive nephrosclerosis: a randomized controlled trial. JAMA.

[42] Lewis EJ, Hunsicker LG, Clarke WR, Berl T, Pohl MA, Lewis JB, Ritz E, Atkins RC, Rohde R, Raz I (2001). Renoprotective effect of the angiotensin-receptor antagonist irbesartan in patients with nephropathy due to type 2 diabetes. N Engl J Med.

[43] Hou FF, Zhang X, Zhang GH, Xie D, Chen PY, Zhang WR, Jiang JP, Liang M, Wang GB, Liu ZR (2006). Efficacy and safety of benazepril for advanced chronic renal insufficiency. N Engl J Med.

[44] Brenner BM, Cooper ME, de Zeeuw D, Keane WF, Mitch WE, Parving HH, Remuzzi G, Snapinn SM, Zhang Z, Shahinfar S (2001). Effects of losartan on renal and cardiovascular outcomes in patients with type 2 diabetes and nephropathy. N Engl J Med.

[45] Solomon SD, Rice MM, A Jablonski K, Jose P, Domanski M, Sabatine M, Gersh BJ, Rouleau J, Pfeffer MA, Braunwald E (2006). Renal function and effectiveness of angiotensin-converting enzyme inhibitor therapy in patients with chronic stable coronary disease in the Prevention of Events with ACE inhibition (PEACE) trial. Circulation.

[46] Susantitaphong P, Sewaralthahab K, Balk EM, Jaber BL, Madias NE (2012). Short- and long-term effects of alkali therapy in chronic kidney disease: a systematic review. Am J Nephrol.

[47] de Brito-Ashurst I, Varagunam M, Raftery MJ, Yaqoob MM (2009). Bicarbonate supplementation slows progression of CKD and improves nutritional status. J Am Soc Nephrol..

[48] Devins GM, Mendelssohn DC, Barre PE, Binik YM (2003). Predialysis psychoeducational intervention and coping styles influence time to dialysis in chronic kidney disease. Am J Kidney Dis.

[49] Robinson BM, Zhang J, Morgenstern H, Bradbury BD, Ng LJ, McCullough KP, Gillespie BW, Hakim R, Rayner H, Fort J (2014). Worldwide, mortality risk is high soon after initiation of hemodialysis. Kidney Int.

[50] Astor BC, Eustace JA, Powe NR, Klag MJ, Fink NE, Coresh J (2005). Type of vascular access and survival among incident hemodialysis patients: the Choices for Healthy Outcomes in Caring for ESRD (CHOICE) Study. J Am Soc Nephrol..

[51] Xue JL, Dahl D, Ebben JP, Collins AJ (2003). The association of initial hemodialysis access type with mortality outcomes in elderly Medicare ESRD patients. Am J Kidney Dis.

[52] Liem YS, Weimar W (2009). Early living-donor kidney transplantation: a review of the associated survival benefit. Transplantation.

[53] Meier-Kriesche HU, Kaplan B (2002). Waiting time on dialysis as the strongest modifiable risk factor for renal transplant outcomes: a paired donor kidney analysis. Transplantation.

[54] Mange KC, Joffe MM, Feldman HI (2001). Effect of the use or nonuse of long-term dialysis on the subsequent survival of renal transplants from living donors. N Engl J Med.

[55] Yee J, Faber MD, Soman SS, Harrington JT, Newman ED (2012). Chronic kidney disease: changing the mean by changing the mien. Great health care: making it happen.

[56] U.S. Renal Data System (2010). USRDS 2010 annual data report: atlas of chronic kidney disease and end-stage renal disease in the United States.

[57] Tangri N, Kitsios GD, Inker LA, Griffith J, Naimark DM, Walker S, Rigatto C, Uhlig K, Kent DM, Levey AS (2013). Risk prediction models for patients with chronic kidney disease: a systematic review. Ann Intern Med.

[58] Tangri N, Stevens LA, Griffith J, Tighiouart H, Djurdjev O, Naimark D, Levin A, Levey AS (2011). A predictive model for progression of chronic kidney disease to kidney failure. JAMA.

[59] Maziarz M, Black RA, Fong CT, Himmelfarb J, Chertow GM, Hall YN. Evaluating Risk of ESRD in the UrbanPoor. J Am Soc Nephrol. 2015;26(6):1434-42. 10.1681/ASN.2014060546 PMC4446879 25475746

[60] Snyder JJ, Collins AJ (2009). Association of preventive health care with atherosclerotic heart disease and mortality in CKD. J Am Soc Nephrol..

[61] Hemmelgarn BR, Manns BJ, Zhang J, Tonelli M, Klarenbach S, Walsh M, Culleton BF (2007). Association between multidisciplinary care and survival for elderly patients with chronic kidney disease. J Am Soc Nephrol..

[62] Wu IW, Wang SY, Hsu KH, Lee CC, Sun CY, Tsai CJ, Wu MS (2009). Multidisciplinary predialysis education decreases the incidence of dialysis and reduces mortality--a controlled cohort study based on the NKF/DOQI guidelines. Nephrol Dial Transplant.

[63] Curtis BM, Ravani P, Malberti F, Kennett F, Taylor PA, Djurdjev O, Levin A (2005). The short- and long-term impact of multi-disciplinary clinics in addition to standard nephrology care on patient outcomes. Nephrol Dial Transplant.

[64] Lacson E, Wang W, DeVries C, Leste K, Hakim RM, Lazarus M, Pulliam J (2011). Effects of a nationwide predialysis educational program on modality choice, vascular access, and patient outcomes. Am J Kidney Dis.

[65] Peeters MJ, van Zuilen AD, van den Brand JA, Bots ML, van Buren M, ten Dam MA, Kaasjager KA, Ligtenberg G, Sijpkens YW, Sluiter HE (2014). Nurse Practitioner Care Improves Renal Outcome in Patients with CKD. J Am Soc Nephrol..

[66] van Zuilen AD, Bots ML, Dulger A, van der Tweel I, van Buren M, Ten Dam MA, Kaasjager KA, Ligtenberg G, Sijpkens YW, Sluiter HE (2012). Multifactorial intervention with nurse practitioners does not change cardiovascular outcomes in patients with chronic kidney disease. Kidney Int.

[67] Richards N, Harris K, Whitfield M, O’Donoghue D, Lewis R, Mansell M, Thomas S, Townend J, Eames M, Marcelli D (2008). Primary care-based disease management of chronic kidney disease (CKD), based on estimated glomerular filtration rate (eGFR) reporting, improves patient outcomes. Nephrol Dial Transplant.

[68] Bayliss EA, Bhardwaja B, Ross C, Beck A, Lanese DM (2011). Multidisciplinary team care may slow the rate of decline in renal function. Clin J Am Soc Nephrol.

[69] Barrett BJ, Garg AX, Goeree R, Levin A, Molzahn A, Rigatto C, Singer J, Soltys G, Soroka S, Ayers D (2011). A Nurse-coordinated Model of Care versus Usual Care for Stage 3/4 Chronic Kidney Disease in the Community: A Randomized Controlled Trial. Clin J Am Soc Nephrol.

[70] Yeoh HH, Tiquia HS, Abcar AC, Rasgon SA, Idroos ML, Daneshvari SF (2003). Impact of predialysis care on clinical outcomes. Hemodialysis international International Symposium on Home Hemodialysis.

[71] Wei SY, Chang YY, Mau LW, Lin MY, Chiu HC, Tsai JC, Huang CJ, Chen HC, Hwang SJ (2010). Chronic kidney disease care program improves quality of pre-end-stage renal disease care and reduces medical costs. Nephrology (Carlton).

[72] Lee W, Campoy S, Smits G, Vu Tran Z, Chonchol M (2007). Effectiveness of a chronic kidney disease clinic in achieving K/DOQI guideline targets at initiation of dialysis--a single-centre experience. Nephrol Dial Transplant.

